# Lectures on Military Surgery

**Published:** 1861-06

**Authors:** E. Andrews

**Affiliations:** Professor of Surgery, Surgeon to the Mercy Hospital; to the Chicago Medical Dispensary, &c.


### CHICAGO MEDICAL EXAMINER

N. S. DAVIS, M. D., Editor.---FRANK W. REILLY, M. D., Ass't Editor.

VOL. II.] JUNE, 1861. [NO. 6.

### Original Communications.

### ARTICLE I.

### LECTURES ON MILITARY SURGERY:

DELIVERED DURING THE SUMMER COURSE OF THE MEDICAL DEPARTMENT OF LIND UNIVERSITY.

By E. ANDREWS, A. M., M. D.,

PROF. OF SURGERY; SURGEON TO THE MERCY HOSPITAL; TO THE CHICAGO MEDICAL DISPENSARY, &C.

### LECTURE SECOND.

Gentlemen:—In my first lecture I gave you an account of the organization of the Medical Department of the United States Army, of the mode of admission into it, and also of the duties of the Surgeon in examining recruits offered for the service.

To-day we treat of more important topics. Many a gallant army, unsmitten by the sword, unopposed by the enemy, has succumbed, and fled in disgrace before invisible foes, whose onset the physician alone knows how to guard or meet. Fortified cities have been taken by pestilence, while besiegers thundered at the gates in vain. Campaigns have been broken without a battle, by cholera and plague; and even when these extreme disasters have not occurred, it has, from all ages, been true, that disease, and not the sword is the chief agent of destruction which thins the ranks of war.

The diseases which thus work the ruin of armies, are of kinds which, by care, may be *effectually prevented;* hence, one of the most important duties of a Military Surgeon is to give advice respecting

### THE SANITARY MANAGEMENT OF THE ARMY.

In war, the curing is of necessity subordinate to the killing department; hence, even the Surgeon-General will not often be allowed to change the march, or locality of troops, for the sake of their health. Nevertheless, the medical officer may give the most important information on this subject to those who direct the troops, and in this way save thousands of lives, which otherwise would have been sacrificed to improper management, unnecessary exposure, and insufficient care. For this reason the Military Surgeon should study *Hygiene* in its every possible application, and become competent to instruct the other officers. He should not only be able to treat the sick and the wounded in the camp, but he should also be competent, at the commencement of the campaign, to point out to the commanders the kinds of disease to be expected, the possible severity of their onset, the places where they will be most prevalent, and the particular months in which they will make their attack. Then, also, he should be competent to show how these evils can be mitigated or prevented; and what particular supplies of food, clothing or shelter are required for that end.

### FOOD AND DRINK OF THE ARMY.

The rank and file of all armies are supplied with an exceedingly simple diet, consisting of little more than bread and meat—the latter generally salt. The daily allowance of food for each man is called his "ration," and is as follows:

| | |
|---|---|
| Bread or flour | 18 oz. |
| Or hard bread | 12 oz. |
| Or corn-meal | 20 oz. |
| Also Beef | 20 oz. |
| Or pork or bacon | 12 oz. |

Besides these there is a small allowance of rice, beans, sugar, coffee, vinegar, salt, soap, and candles. The articles of diet, therefore, are limited in number, hence, if there is any failure in the quality or quantity of the rations, it tells with ruinous effect on the health and spirits of the soldiers. It is the duty, therefore, of the commander in providing for the health of the army, to see that the food of the men shall in no case be scanty or bad. So far as possible, fresh meat should be given out instead of salt. A body of men kept exclusively upon salt food, will inevitably be attacked with *scurvy.* This seems not to be sufficiently understood by many military men. The impression is, that scanty diet, hard toil, and absence of vegetables are the principal causes of scurvy, and not salt food. This is an error. *Scurvy is the specific effect of an excess of salt in the system, just as much as mercurialization is of mercury, or iodism is of iodine.* Starvation, hard toil, and absence of vegetables, all combined, will not produce it, if the little food that is eaten be *fresh.* This was well shown in Dr. Kane's famous expeditions to the north in search of Sir John Franklin. Dr. Hayes, who was of the party, informs me that, though the Esquimaux often suffer extremely from starvation, cold, and other hardships, they never have scurvy, because the little food they do get in times of scarcity is *fresh meat;* while the crews of vessels which frequent these waters, living on salt food alone, are afflicted beyond measure. At one time Dr. Kane's command was divided into two parties, one of which remained on the ship with plenty of *salt* food, and a good shelter, with a supply of fuel. The other made an expedition on the ice, slept out of doors, got out of provisions, and had to live solely on some very scanty supplies of fresh walrus-meat, obtained from the Esquimaux. In this case the sheltered and well-fed party in the ship, living on salt food, was smitten with the scurvy; while the famished and overworked company on the ice, having only fresh flesh diet, without any vegetables at all, were entirely free from the disease.

If, then, the army is kept exclusively on salt food, and the men have no opportunity of getting anything else, scurvy will inevitably appear; nor will the Surgeon be able to prevent it by doling out little glasses of lemon juice, or other supposed specifics for the disease.

If the circumstances of the campaign prevent, for any long time, the obtaining of fresh food, the soldiers should be taught the art of freshening their meat before cooking it, by steeping it in cold water; and at the earliest possible moment, the continuity of the salt supply should be interrupted, by the slaughter of fresh beeves. Salt meat may be well freshened by cutting it into pieces of about four ounces and letting it stand in the kettles over night, covered with cold water. The water will extract most of the salt and leave the meat more suitable for cooking. In border warfare upon the western plains, a very excellent resource is the *pemmican* of the Indians and the French traders. This consists of buffalo meat dried fresh and beaten up with the fat. It is portable, keeps perfectly any length of time, and may be kept on hand for an occasional meal when other fresh supplies fail. Of course, all opportunities should be embraced to supply the men with vegetables; they are very important to health, but I wish you, gentlemen, to understand that the chief cause of scurvy is *not the absence of vegetables*, but the presence in excess of chloride of sodium. The modes of cooking practiced by the soldiers have a considerable effect on their health, and the Surgeon should be competent to give complete and practical directions upon the subject. In the Crimean war, the famous cook, Soyer, was sent out by the government, and succeeded in effecting great improvements in this department. The following are some of his directions:

### SOUP FOR FIFTY MEN.

"Put into the boiler, 7½ gallons of water; fifty pounds of beef or mutton; three pounds of rice; eight pounds of fresh vegetables; ten tablespoonfuls of salt; one tablespoonful of pepper. Simmer three hours, and serve. Skim off the fat, which, when cold, is an excellent substitute for butter.

### PLAIN IRISH STEW FOR FIFTY MEN.

Cut fifty pounds of mutton into pieces of a quarter of a pound each; put them into the pan; add eight pounds of onions, twelve pounds of whole potatoes, eight tablespoonfuls of salt, three tablespoonfuls of pepper; cover all with water, giving about half a pint to each pound; then light the fire. An hour and a half of gentle boiling will make a most excellent stew. Mash some of the potatoes to thicken the gravy, and serve. Fresh beef, veal, or pork, will also make a good stew. Beef takes two hours doing.

This I know to be a most excellent dish. It can always be prepared upon the march, in time for the men's dinner or supper.

FRENCH BEEF SOUP—CAMP FASHION.

Put in the camp kettle, six pounds of beef, cut into two or three pieces, bones included; three-quarters of a pound of plain, mixed vegetables, as onions, carrots, turnips, celery, as can be obtained, or three ounces of desiccated vegetables; three teaspoonfuls of salt, one of pepper, one of sugar; eight pints of water. Let it boil gently for three hours; remove some of the fat, and serve.

The addition of half a pound of bread, cut in slices, or one pound of broken biscuit, well soaked in the broth, will make a very nutritious soup. This is enough for six or eight people. The quantity required for a company is easily calculated.

STEWED SALT BEEF AND PORK FOR ONE HUNDRED MEN

Put in a boiler of well soaked beef, thirty pounds, cut in pieces of a quarter pound each; twenty pounds of pork, cut up; one and a half pounds of sugar; eight pounds of onions, sliced; twenty-five quarts of water; four pounds of rice. Simmer gently for three hours; skim the fat off the top, and serve.

If soup is not made, the beef may still be boiled, but in a different way. Soup should be made by beginning the process with cold water at first, and then simmer at 160° till done.

The general rule is, that roasting is to be done quickly, and boiling slowly.

In roasting, the loss of beef is nineteen, mutton twenty-four, but their chemical constitution is not changed. The loss is made up of water evaporated, and fat melted and mixed with gravy. But in boiling, the loss, though less in amount, is more serious in quality; it is made up of fat, gelatine and water, with some albumen and hematosine; and if salt is used, potash is likewise lost. Therefore the broth should be eaten.

The bean soup, made by soldiers in the army, is the best I have ever eaten. They prepare it by soaking the beans all night. The ration of pork is boiled in it, and any fresh or desiccated vegetables they can procure. Potatoes and onions are great favorites with them. It is seasoned to the taste with salt and pepper."

In tropical countries, the men occasionally suffer from improper indulgence in various fruits; but as a general rule, a large army will not be able to get enough of them to be in any serious danger from excesses of this kind.

The supply of suitable water for drinking is also important. If encamped by marshy ground, the men should be prevented from drinking the deleterious vegetable infusion, dipped up from the swamp. Good water should, if necessary, be brought even from a distance by teams. In the southern parts of our great west, the inhabitants make much use of rain water for drink, believing it to be healthier in their country, than the water of the springs and wells. It will not often be the case that armies, however, can avail themselves of this resource, though small detachments may find it convenient and safe to follow the custom of the country in that respect, and supply themselves from cisterns. The water of the Mississippi will, of necessity, furnish the drink of great bodies of men during the present war. From the confluence of the Missouri downwards, this stream presents a foul appearance, highly alarming to men who are accustomed to drink from the crystal springs of the north. The stream has the exact color of the water which flows in the gutters of a dusty wagon road after a thunder storm. If a pailful of it is dipped up, it will deposit a half an inch of mud upon its bottom in settling. The unhealthy appearance of this water is, however, a delusion. The sediment in it is mostly insoluble mineral matter, which is deposited upon filtering or settling, and there is actually much less in it that is unwholesome than in many hillside springs. The inhabitants of the banks of this great river, after full experience, pronounce its muddy waters a perfectly safe drink.

Coffee has been highly praised as a daily drink for soldiers. I do not think it has any sanitary advantage over water, except that it gives an air of comfort to the camp, and makes the soldier more cheerful at his meals. Alcoholic liquors have long been a part of the regular supplies of our army, but they have latterly been discontinued, except in cases of unusual exposure, and coffee and tea are substituted in their place. In cold climates it is probable that the daily use of alcohol is less injurious than in the tropics; but in warm regions there is no doubt that the daily dram causes many a stout soldier to succumb to the deadly climate. Men who drink habitually are not as hardy under climatic exposure as others. Almost all the cases of sun-stroke occur to habitual drinkers; and in every other climatic disease they are the first victims. If the Surgeon's professional opinion, therefore, is required, it should be to the purport that alcohol in a hot climate is deleterious, and that the men are physically better without it. If a stimulant is demanded, coffee is undoubtedly to be preferred.

On a march, the canteens of the men should be frequently filled with water, in order that by often sipping it they may be prevented from suffering excessive thirst, and not be under a temptation on arriving at a stream to rush in and drink to a fatal excess. They should be cautioned after great and exhausting efforts against this imprudence.

### CLOTHING.

No arbitrary rules can be given under this head. The clothing should be adapted to the climate. In cold regions warmth is to be secured, and in the tropics coolness sought after. The stiff adherence in a climate like that of India, to a dress, devised for warmth in England, is absurd; and a similar error should not be committed in this country. In the hot summer of our gulf States, the usual dress of the soldier should be of thin porous woolen, loose and free, and not absurdly buttoned to the chin. Heavy caps, hats and helmets may be tolerated occasionally on parade, but should not appear in battle, nor on a march. The soldier should have a single substantial blanket, strong shoes, and woolen stockings. The hat, whatever it is, should be porous and allow free ventilation between it and the top of the head. On marches, and in battles undertaken in hot weather, a wet handkerchief placed within the hat keeps up an evaporation and goes far to protect the person from sun-stroke. It is desirable that some other shelter from the sun's rays should be attached to the dress. The Arabs of the desert always wear a sort of cape or veil covering the entire scalp and back of the neck. The British soldiers in India found it necessary to devise a similar thing, and probably it will be required in this country also. A suitable cap-cover may be made of any white or light colored fabric, which does not, like black, grow hot in the sun. It should cover the whole top, sides and back of the head, and hang down as far as the shoulders behind. This will be found to materially diminish the mortality from sun-stroke.

The proper dress for a soldier is a difficult problem in some respects; and the Surgeon will seldom see his own wishes in the matter, put in execution by the government; nevertheless, he should be prepared to state clearly the true principles involved in the hygiene of clothing.

